# Index Catalogue of the Library of the Surgeon-General’s Office

**Published:** 1887-04

**Authors:** 


INDEX CATALOGUE OF THE LIBRARY OF THE SURGEON GENERAL'S OFFICE, U. S. ARMY. *Vol. VII. Insignares, Leghorn. Pp. [100] and 959.*

In his letter to the Surgeon-General which accompanies this volume, the compiler (Dr. J. S. Billings) says: "This volume includes 14,688 author-titles, representing 5,987 volumes and 12,372 pamphlets. It also includes 6,371 subject-titles of separate books and pamphlets and 34,903 titles of articles in periodicals." It also includes a complete alphabetical list of abbreviations of titles of medical periodicals employed in the index-catalogue.

It is impossible in a notice of this volume to convey an adequate idea, either of the magnitude or value of the work which it represents. It is in keeping with the uniform excellence and completeness of the other volumes of the series, which have been issued from the Surgeon-General's office. This index-catalogue has become a necessity in looking up the literature of any given medical subject, and it is very highly appreciated by those who have learned to know its value.

